# Low-Temperature Terpolymerizable Benzoxazine Monomer Bearing Norbornene and Furan Groups: Synthesis, Characterization, Polymerization, and Properties of Its Polymer

**DOI:** 10.3390/molecules28093944

**Published:** 2023-05-07

**Authors:** Yin Lu, Yaliang Peng, Yi Yang, Jiahao Liu, Kan Zhang

**Affiliations:** 1 Research School of Polymeric Materials, School of Materials Science and Engineering, Jiangsu University, Zhenjiang 212013, China; luy@stmail.ujs.edu.cn (Y.L.); yaliangpeng@163.com (Y.P.); a19851781037@163.com (Y.Y.); 2 School of Mechanical Engineering, Jiangsu University, Zhenjiang 212013, China; liujiahaohaohao@outlook.com

**Keywords:** benzoxazine, norbornene, furan, thermosets, thermal stability

## Abstract

There is an urgency to produce novel high-performance resins to support the rapid development of the aerospace field and the electronic industry. In the present work, we designed and consequently synthesized a benzoxazine monomer (***o*HPNI-fa**) bearing both norbornene and furan groups through the flexible benzoxazine structural design capability. The molecular structure of ***o*HPNI-fa** was verified by the combination characterization of nuclear magnetic resonance spectrum, FT-IR technology, and high-resolution mass spectrum. The thermally activated terpolymerization was monitored by in situ FT-IR as well as differential scanning calorimetry (DSC). Moreover, the low-temperature-curing characteristics of ***o*HPNI-fa** have also been revealed and discussed in the current study. Furthermore, the curing kinetics of the ***o*HPNI-fa** were investigated by the Kissinger and Ozawa methods. The resulting highly cross-linked thermoset based on ***o*HPNI-fa** showed excellent thermal stability as well as flame retardancy (T_d10_ of 425 °C, THR of 4.9 KJg^−1^). The strategy for molecular design utilized in the current work gives a guide to the development of high-performance resins which can potentially be applied in the aerospace and electronics industries.

## 1. Introduction

The use of polymer matrix composites in aircraft design provides significant benefits, including increased fuel efficiency, reduced weight, improved damage tolerance, and enhanced durability. These materials are also more resistant to fatigue and corrosion and can withstand high temperatures and extreme environments, making them ideal for use in aerospace applications [1,2,3]. Through the advancements in material science and manufacturing technology, engineers have been able to design and produce polymer matrix composites with tailored properties specific to their intended applications. This has allowed for the development of aircraft that are faster, more maneuverable, and more efficient than ever before [4,5]. Furthermore, the use of polymer matrix composites in aerospace manufacturing has also contributed to the reduction of the environmental impact of air travel. The reduced weight of aircraft has led to decreased fuel consumption, resulting in lower greenhouse gas emissions and a more sustainable aviation industry. Overall, the application of polymer matrix composites in aerospace manufacturing has been a key factor in the development of more advanced and efficient aircraft, contributing to the safety, reliability, and sustainability of the aviation industry [6,7,8,9].

**Scheme 1:**
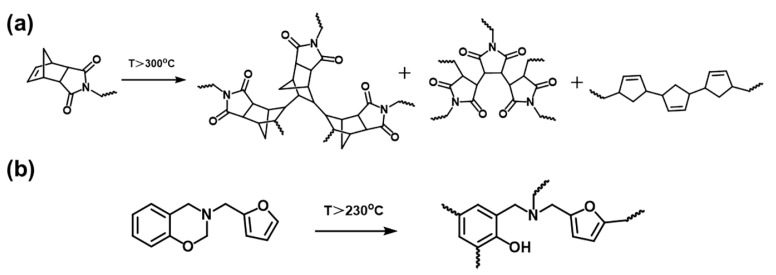
Polymerization behavior of the nadic-end-capped oligomer (**a**) and furan-based benzoxazine (**b**).

In recent years, the development of advanced materials has been a major focus in the aerospace industry. Structural thermosets such as PMR families are an example of new materials that are being used in aerospace and military applications. PMR-15 is a polyimide-based thermosetting resin that contains norbornene functionality. It is renowned for its exceptional thermal stability, chemical resistance, and mechanical strength, and its high resistance to shock and vibration makes it an ideal material for use in aerospace and military technology [10,11].PMR-15 also exhibits a lightweight characteristic and possesses a low coefficient of thermal expansion, which makes it suitable for use in high-temperature environments [12,13,14]. However, a major drawback of norbornene end-capped polymers, including PMR-15, is that they require a high polymerization temperature in excess of 300 °C to generate the cross-linked network that is necessary for their thermal and mechanical properties (Scheme 1a) [15]. Despite these challenges, recent studies have shown that the curing temperature of norbornene can be significantly reduced by incorporating it into smart *ortho*-benzoxazines. Smart *ortho*-benzoxazines are a type of monomer that can be polymerized at relatively low temperatures, typically between 180 °C and 220 °C. The resulting polymers exhibit excellent thermal stability, chemical resistance, and mechanical performance that are comparable to those demonstrated by PMR-15 and other norbornene-based materials. Moreover, these polymers possess a low coefficient of thermal expansion, making them a suitable choice for high-temperature aerospace applications [15,16,17]. The use of smart *ortho*-benzoxazine polymers could offer significant advantages over other thermosets in aerospace and military applications. They are lightweight, easy to process, and can be easily incorporated into complex structures. Moreover, their ability to be cured at low temperatures would reduce manufacturing costs and allow them to be used in a wider range of applications [15,16,17]. The aerospace industry is always seeking new advanced materials that can improve the performance, durability, and safety of aerospace technology. The development of smart *ortho*-benzoxazines is exciting because they offer the potential for high-performance materials that can be cured at low temperatures, making them cost-effective and easy to incorporate into complex structures.

As a novel emerging structural resin, benzoxazine resin has demonstrated various incomparable advantages [18,19,20,21,22]. The most appealing aspect of benzoxazine resin is its flexible structure design capability as well as the high-performance properties of its polymer [23,24,25]. Recently, a new series of benzoxazine resins with high-performance characteristics, namely, *ortho*-imide functional benzoxazines, have been reported and systematically investigated by our research group [26,27]. Interestingly, these *ortho*-functional polybenzoxazines with rigid imide moiety display a number of desirable characteristics, including unexpected low-temperature curing, higher values of *T*_g_, and improved thermal stability, compared to conventional polybenzoxazines generated from *para*-functional counterparts [28,29]. In addition, the synthesis of *ortho*-functional polybenzoxazines with additional chemical groups, such as alkynyl, cyan group, and phthalonitrile units, have also been reported [15,27,30,31]. Previous studies relating to *ortho*-imide functional benzoxazines indicate the possibility of designing high-performance thermosets based on smart benzoxazine resins.

The world’s fossil resources are facing depletion at an alarming rate, making the switch to renewable sources and the development of corresponding technologies a necessity. This has led to a surge of interest in sustainable materials science, which has seen significant advances in the development of new polymers with desired and controllable properties. However, the practical application of benzoxazine resins, especially on an industrial scale, faces similar challenges to most other commercialized polymeric materials in terms of the availability of its raw materials. In response, researchers have been working tirelessly to synthesize sustainable benzoxazine through biomass resources, resulting in the creation of furan-based benzoxazine, which has made up a significant proportion of all reports [32,33,34,35,36,37,38]. Despite the complex mechanism behind the ring-opening polymerization (ROP) of benzoxazine, research has shown that the improvement in the characteristics of furan-containing polybenzoxazines can be associated with an increase in the number of reactive centers, leading to a higher degree of cross-linking [36,39,40]. Furthermore, bio-benzoxazine monomers have demonstrated relatively lower curing temperatures, which make them even more attractive for industrial use. The development of sustainable benzoxazine has also presented an opportunity for researchers to actively pursue a more environmentally friendly future. The use of renewable resources to synthesize the bio-benzoxazine monomer has provided a promising alternative to traditional fossil-based systems. As such, research advances in the field of sustainable materials science, specifically in the development of benzoxazine resins, are a crucial step in ensuring a sustainable future for the planet. In light of this, it is of the utmost importance for the research community to continue its efforts in exploring sustainable alternatives and to make significant strides towards overcoming the challenges associated with a practical industrial application of sustainable materials, and furan-based materials might be a promising choice. The typical curing behavior of furan-based benzoxazine (**PH-fa**) is proposed in Scheme 1b [34,41].

From all the above findings, we are inspired to incorporate both norbornene and furan structures into *ortho*-benzoxazine to obtain high-performance thermosetting resin with low curing temperature and excellent properties. Herein, ***o*HPNI-fa**, a new benzoxazine monomer, has been designed and synthesized. In order to obtain a thorough grasp of the numerous polymerization processes of the recently discovered norbornene–benzoxazine–furan thermosetting system, two benzoxazine monomers, which only contain the norbornene or furan groups, were also prepared as counterparts. More significantly, the obtained ***o*HPNI-fa** provides a sample to investigate in depth the structural changes during the polymerization of a terpolymerizable monomer. Furthermore, this work also provides a very compelling illustration for actual applications, particularly for high-performance regions demanding non-flammable polymeric matrices.

## 2. Results and Discussion

### 2.1. Design, Synthesis, and Structural Characterization of the Benzoxazine Monomer

Based on thorough investigations of *ortho*-functional benzoxazine, *ortho*-imide-based benzoxazine was chosen as the central structure due to its advantage in high-yield synthesis [26]. We then sought out ways to incorporate the norbornene and furan groups into the benzoxazine monomer structure. Consequently, an *ortho*-norbornene phenol (***o*HPNI**) was chosen as the phenol source, and furfurylamine was selected as the amine source to conduct a Mannich condensation. As a result, we were successful in developing and creating a benzoxazine monomer containing both norbornene and furan functionalities.

**Figure 1:**
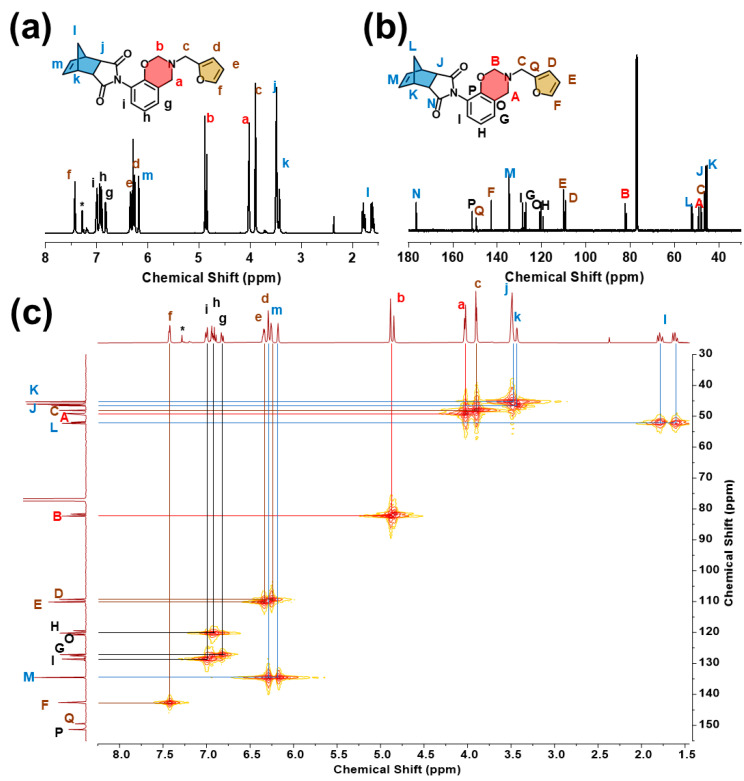
^1^H (**a**), ^13^C (**b**), and ^1^H-^13^C HMQC (**c**) NMR spectra of ***o*HPNI-fa** in CDCl_3_ (*: CDCl_3_, lowercase: H proton, capital: C).

The molecular structure of novel monomer containing both norbornene and furan functionalities was first characterized by NMR technology, which is shown in Figure 1. ^1^H NMR spectra of ***o*HPNI-fa** in CDCl_3_ is illustrated in Figure 1a. The -CH_2_- group in the oxazine ring is responsible for two singlet resonances with similar intensity for 1,3-benzoxazines. On the other hand, two doublets are seen for ***o*HPNI-fa** at 4.02 and 4.04 ppm and 4.85 and 4.89 ppm, which can be credited to Ar-CH_2_-N and -O-CH_2_-N, respectively. Since the norbornene endcap and imide five-membered ring is on a different plane, which permits the occurrence of atropisomerism in ***o*HPNI-fa**, so the two peaks occurred in the form of a typical branching resonance [27]. Moreover, the three protons that come from the furan group have respective concentrations of 7.43, 6.34, and 6.27 ppm. The protons of the double bond in the norbornene group are responsible for the singlet at 6.18 ppm. In Figure 1b, the ^13^C NMR spectrum of ***o*HPNI-fa** is also shown. The typical four carbons in the furan structure are responsible for resonances at 109.3, 110.1, 142.6, and 149.3 ppm, respectively. In addition, the -C=C- bond in the norbornene group causes the signal at 134.5 ppm. The typical carbon resonances for the oxazine ring, Ar-O-CH_2_-N- and Ar-CH_2_-N-, are found at 82.2 ppm and 49.0 ppm, respectively. Moreover, the typical aromatic carbon in the furan ring, -N-CH_2_-fur, can be found at 47.9 ppm. The findings from the ^1^H-^13^C HMQC spectrum (Figure 1c) confirm the precise distribution for the carbons in the norbornene, furan, and oxazine structure in ***o*HPNI-fa**. All the above findings confirm the successful incorporation of the furan and norbornene groups into the benzoxazine monomer.

**Figure 2:**
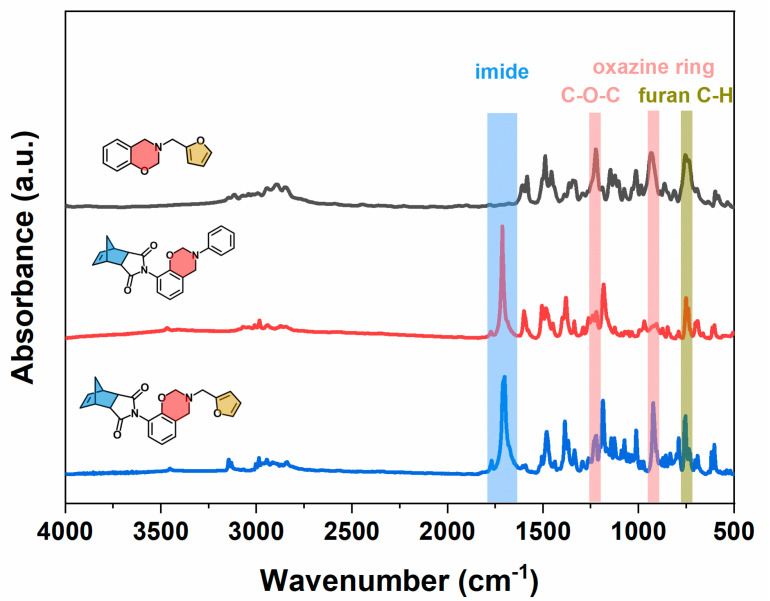
FT-IR spectra of **PH-fa**, ***o*HPNI-a**, and ***o*HPNI-fa**.

FTIR analysis was conducted to further validate the chemical structures of ***o*HPNI-fa**, and the resulting spectra of **PH-fa**, ***o*HPNI-a**, and ***o*HPNI-fa** are displayed in Figure 2. The oxazine ring, norbornene, and furan are all presented in the current synthesized benzoxazine monomer, which is supported by the marked absorption bands in Figure 2. The distinctive band around 755 cm^−1^ is undoubtedly caused by out-of-plane in-phase wagging of the C-H bond in the furan structure, and bands at 1501 and 1081 cm^−1^, which are credited to the C=C stretching and C–O antisymmetric stretching in furan, can also be observed [42]. Moreover, the imide group is allocated to the distinctive bands at 1769 and 1701 cm^−1^. The asymmetric and symmetric stretching modes of the imide I (C-C(C=O)-C) are responsible for the usual doublet bands at 1769 and 1701 cm^−1^. The axial stretching of the C-N bond is attributed to the band at 1385 cm^−1^. The C-O-C asymmetric stretching mode-induced band at 1226 cm^−1^ further supports the idea that the monomer contains oxazine ring aromatic ether. At 925 cm^−1^, the recognizable oxazine-related pattern can be found [43].

The HR-MS spectrum is presented in Figure S1. The pronated species of ***o*HPNI-fa** was found to be 377.1494, which is very close to the calculated theoretical mass of ***o*HPNI-fa**. Based on the aforementioned spectroscopic methods, it has now been proven that the current work successfully synthesized the newly designed benzoxazine monomer, ***o*HPNI-fa**.

### 2.2. Polymerization Behaviors of **o*HPNI-fa***

**Figure 3:**
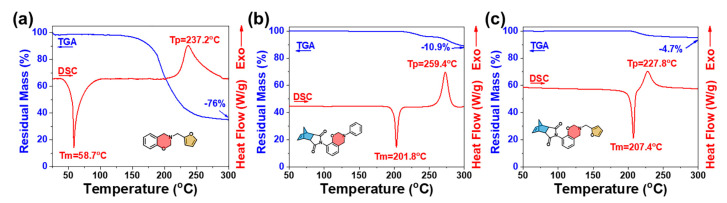
DSC-TG thermograms of **PH-fa** (**a**), ***o*HPNI-a** (**b**), and ***o*HPNI-fa** (**c**).

DSC-TG was used to investigate the thermally stimulated polymerization behavior of ***o*HPNI-fa**. It is well known that the purity of benzoxazine samples may result in different DSC thermograms of the same benzoxazine sample. Thus, we only used very pure samples of benzoxazine resin in the current study, which will show reliable mechanisms in terms of thermally activated ring-opening polymerization of benzoxazines. The DSC-TG curves of **PH-fa**, ***o*HPNI-a**, and ***o*HPNI-fa** are given in Figure 3. **PH-fa**, a mono functional benzoxazine monomer, is synthesized from phenol and furfurylamine, whereas ***o*HPNI-a** is intended to be a monofunctional benzoxazine with solely norbornene group. The extremely sharp endothermic peaks that appear on each thermogram in Figure 3 demonstrate the excellent purity of the benzoxazine monomers. ***o*HPNI-a** has a highest polymerization temperature centered at 259.4 °C, whereas **PH-fa** displays a lower polymerization temperature, at 237.2 °C, than PH-a (a benzoxazine monomer produced by Mannich condensation of phenol and aniline), by 35 °C, which clearly indicates that the addition of furan into the benzoxazine monomer can greatly facilitate the polymerization of the oxazine ring [44]. Moreover, ***o*HPNI-fa** shows the lowest polymerization temperature centered at 227.8 °C. Both **PH-fa** and ***o*HPNI-a** have much higher polymerization temperatures than the benzoxazine with norbornene and furan substituents. In ***o*HPNI-fa**, three sets of thermally stimulated polymerization should occur, including cross-linking of the furan group, olefin polymerization, and typically ring-opening of oxazine ring. Nevertheless, there is only a sole exothermic peak that can be seen for ***o*HPNI-fa**, as shown in the DSC program displayed in Figure 3c, which might be explained by overlapping heat events that occur during the polymerization. The DSC findings suggest the presence of a self-catalytic polymerization process that may be brought about by interactions between benzoxazine, furan, and norbornene, since both furan and norbornene groups are included in a single benzoxazine structure.

Figure 3 also compares the TGA thermograms of three monomers (**PH-fa**, ***o*HPNI-a**, and ***o*HPNI-fa**). Prior to the cross-linking of **PH-fa**, a significant quantity of evaporation (up to 76% of the original mass) can be seen, while no clear weight decrease for ***o*HPNI-a** or ***o*HPNI-fa** was observed. Little weight decrease was found for ***o*HPNI-a** (10.9%) and ***o*HPNI-fa** (4.7%) before the polymerization temperature, which is substantially lower than that of **PH-fa**. The heat stability of benzoxazine resins throughout the polymerization processes has therefore been greatly enhanced with the incorporation of the norbornene and furan structure. Notably, ***o*HPNI-a** and ***o*HPNI-fa** possess smaller weight reductions than many other reported monofunctional benzoxazines [45,46].

**Figure 4:**
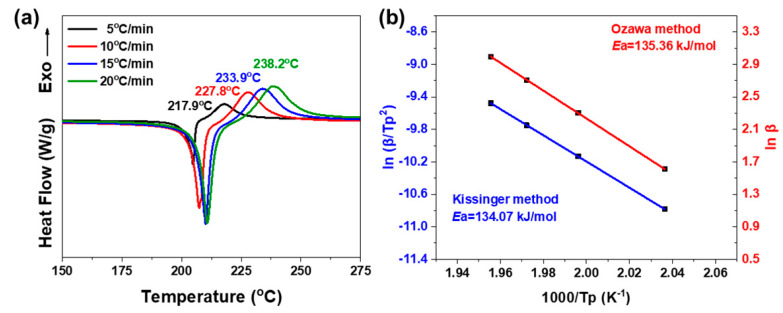
(**a**) DSC curves of ***o*HPNI-fa** at different heating rates. (**b**) Representations of Kissinger and Ozawa methods for the calculation of activation energy for ***o*HPNI-fa**.

According to the theories proposed by Kissinger and Ozawa [47,48], Figure 4 depicts the plots of ln(β/Tp^2^) and ln(β) against 1/Tp for ***o*HPNI-fa**. The plots for **PH-fa** and ***o*HPNI-a** are also shown in Figures S2 and S3. The activation energies (Ea) are estimated and are shown in Table 1. ***o*HPNI-fa** has a substantially greater activation energy than **PH-fa** and **oHPNI-a**. Despite the fact that ***o*HPNI-fa** goes through a terpolymerization process that is different to the dual copolymerization process for **PH-fa** and ***o*HPNI-a**, this does not imply that ***o*HPNI-fa** is more difficult to activate into the polymerization process.

**Figure 5:**
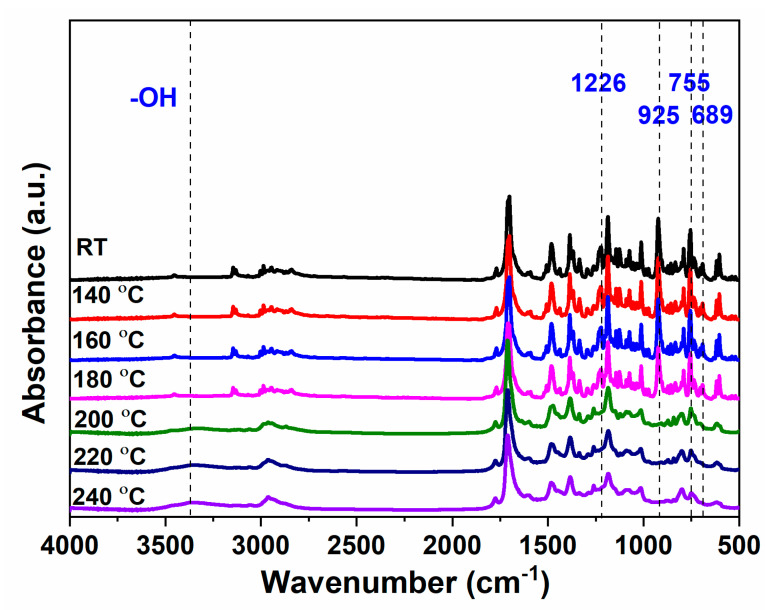
In situ FT-IR spectra of ***o*HPNI-fa** after various thermal treatments.

**Scheme 2:**
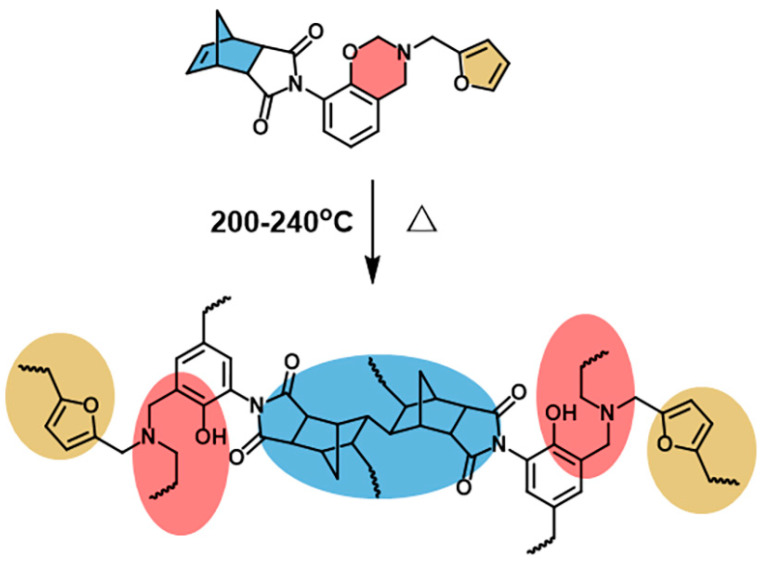
Proposed thermal behaviors of ***o*HPNI-fa**.

Figure 5 depicts the *in situ* FT-IR spectra of ***o*HPNI-fa** following various heat treatments. The thermal behavior of benzoxazine monomer can be monitored using the distinctive absorption bands at 1222 and 924 cm^−1^ which are contributed by the C-O-C antisymmetric stretching mode and benzoxazine-related mode, respectively. When the temperature rises, both bands become weaker until they completely vanish at 220 °C. Additionally, the distinctive bands of the internal C-H in the furan structure at 750 cm^−1^ decline when temperature rises to 200 °C and completely vanish at 220 °C, which indicates the complete cross-linking of the furan structure. Moreover, the characteristic band of the C=C group at 689 cm^−1^, which is due to the out-of-plane bending motion of olefinic -C-H bands, reduces considerably from 180 °C to 220 °C, indicating the simultaneous cross-linking of the norbornene structure. Furthermore, the broadband in the range of 3500–3200 cm^−1^, which is a result of the -OH band, also appears as the heating temperature increases. According to the DSC data, the norbornene polymerization temperature in ***o*HPNI-fa** is substantially lower than it is in **PH-fa** and ***o*HPNI-a**, which indicates that three processes, including cross-linking of both the furan structure, oxazine ring, and norbornene functionalities, are active during the exotherm in the DSC profile of ***o*HPNI-fa**. The furan group can thus speed up the additional cross-linking of the C=C band as well as the polymerization of the oxazine group. The results of this study suggest that additional polymerization reactions from the furan and norbornene functionalities have a great impact on the cross-linking events in forming poly(***o*HPNI-fa**). The proposed terpolymerization process of ***o*HPNI-fa** is shown in Scheme 2.

### 2.3. Thermal Stability and Anti-Flame Properties of Highly Cross-Linked Thermoset

**Figure 6:**
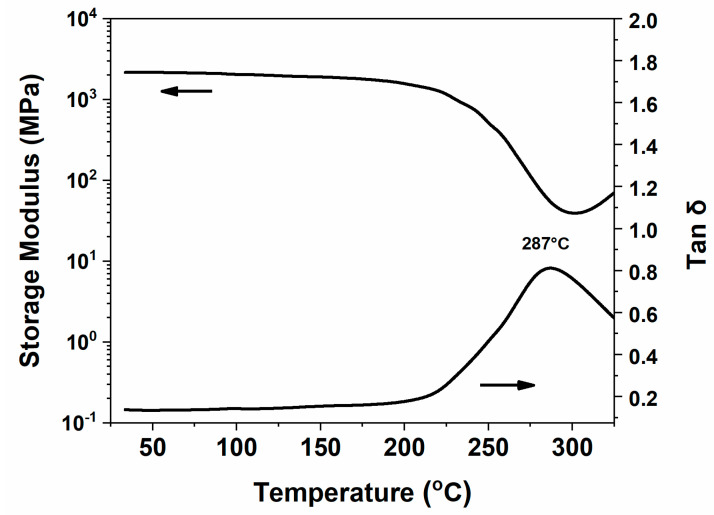
DMA result of poly(***o*HPNI-fa**).

The results obtained from the DMA test are shown in Figure 6. The *T*_g_ (glass transition temperature) of **poly(*o*HPNI-fa)** was found to be 287 °C, which presented as the peak from the curve of tan δ. The copolymerization of norbornene, benzoxazine, and the furan structure bring about the well cross-linked frameworks, resulting in such an attritive glass transition temperature of **poly(*o*HPNI-fa)**.

**Figure 7:**
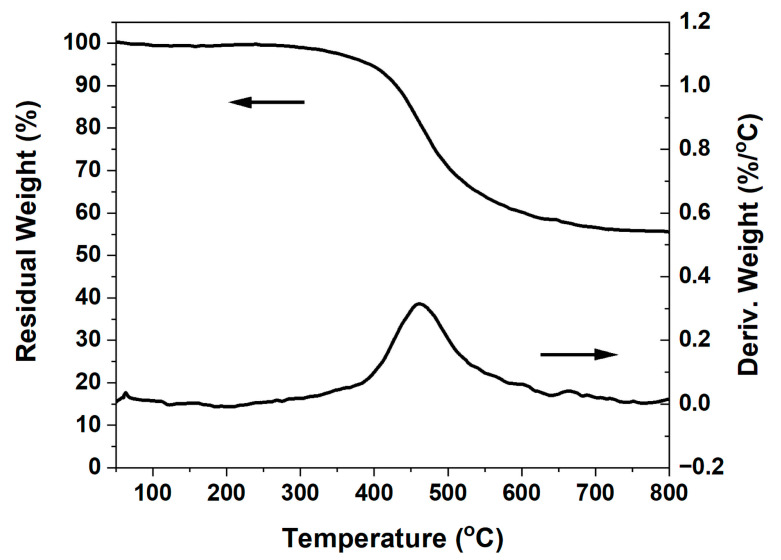
The thermogravimetric analysis and derivative weight loss curves of **poly(*o*HPNI-fa)**.

The thermogravimetric analysis (TGA) performed under N_2_ was used to examine the thermal stability of highly cross-linked thermosets. Figure 7 displays the TG and DTG curves. Table 2 summarizes the data of the char yield values at 800 °C (Yc) and the temperatures with weight loss of 5% (T_d5_) and 10% (T_d10_) during the TGA measurements. There is a high Yc value of 56% for the derived polybenzoxazines. When it comes to conventional polybenzoxazines, the initial weight-loss stage typically occurs between 250 °C and 400 °C, which is linked to flaws in the polybenzoxazine networks that result in the partial breakdown of terminal groups. However, in terms of **poly(*o*HPNI-fa)**, the T_d5_ value is found to be as high as 395 °C. The maximum recorded weight loss is approximately 480 °C. **Poly(*o*HPNI-fa)** has a remarkable thermal stability as a result of its densely cross-linked networks that are contributed by copolymerization of the norbornene, benzoxazine, and furan structures. Moreover, **poly(*o*HPNI-fa)** displays a char yield (Yc) up to 56%. The high char yield and initial decomposition temperature point to the remarkable thermal stability of **poly(*o*HPNI-fa)**.

The LOI (limiting oxygen index) value, which was determined from the Yc via the van Krevelen equation to quantify the flame retardancy of polybenzoxazines using TGA measurements, is shown as follows [49].

LOI (%) = 0.4Yc + 17.5(1)

The calculated LOI value for **poly(*o*HPNI-fa)** based on the above TGA result was 39.9, which far exceeds the self-extinguishing standard (LOI > 28) [50]. This implies that the highly cross-linked resin obtained in the current work may have a promising flame-retardant property.

**Figure 8:**
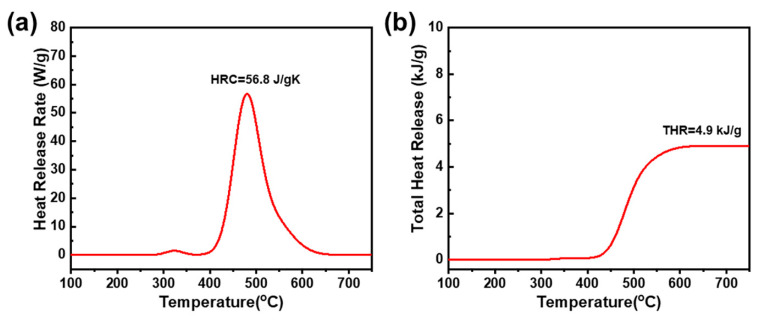
Heat release rate (**a**) and total heat release (**b**) versus temperature for **poly(*o*HPNI-fa)**.

From the calculated LOI value mentioned above, we further investigated the flame-retardant property of **poly(*o*HPNI-fa)**. As shown in Figure 8, **poly(*o*HPNI-fa)** was discovered to have HRC and THR values of 56.8 Jg^−1^K^−1^ and 4.9 kJg^−1^, respectively. Better flame retardancy is often indicated by lower HRC and THR values. Polymers can be considered non-ignitable materials that satisfy the stringent specifications set out by the FAA (Federal Aviation Administration) to be used in commercial airplanes when the HRC value is less than 100 Jg^−1^K^−1^ [51]. Because of their extremely low flammability, thermosets based on the terpolymerized polybenzoxazine offer tremendous application potential in both the aviation and electronic industries according to the MCC results. Table 2 summarizes the thermal and anti-flame characteristics of **poly(*o*HPNI-fa)**.

## 3. Materials and Methods

### 3.1. Materials

The following were obtained from Aladdin Reagent, China: 4-aminophenol (98%), endo-5-norbornene-2,3-dicarboxylic anhydride, paraformaldehyde (99%), furfurylamine (99%), and sodium hydroxide (NaOH) (98%). Toluene and ethanol were purchased from Shanghai First Chemical Co., Shanghai, China. All the above chemicals were used as received. ***o*HPNI** and ***o*HPNI-a** were synthesized according to the previous report [17]. **PH-fa** was synthesized according to our recent report [34].

### 3.2. Characterization

The nuclear magnetic resonance spectra (^1^H NMR, ^13^C NMR, and 2D ^1^H-^13^C HMQC) were recorded with the Bruker AVANCE II 400 MHz NMR spectrometer. Chloroform-*d* was used as solvent in all the NMR tests, the average number of transients was 64, and the total number of transients was 1024. Infrared spectra were collected using a Fourier transform infrared (FT-IR) spectrometer (Nicolet Nexus 670), using KBr technology to supplement the molecular studies of NMR. The measurements were made with a 4 cm^−1^ resolution. An Elementar Vario EL-III was used to record the elemental analysis for the newly obtained monomer. High-resolution mass spectrometry (HR-MS) analysis was carried out on a Thermo Scientific Q Exactive Orbitrap. DSC thermograms were obtained using a NETZSCH differential scanning calorimeter (Model STA449C) under a nitrogen flow rate of 60 mL/min, with different temperature ramps rates of 5, 10, 15, and 20 °C/min. With a NETZSCH DMA/242E analyzer, dynamic mechanical analysis (DMA) was performed using a tension mode with a frequency of 5 Hz under air. A 3 °C/min heating rate was chosen for DMA measurements. Thermogravimetric analysis (TGA) was performed on a NETZSCH STA449 thermogravimetric analyzer while it was being purged with nitrogen at a flow rate of 40 mL/min. The heating rate for TGA was 10 °C/min. Heat release rates (HRR, W/g) of the obtained polymer were measured with a microscale combustion calorimeter (FTT-FAA-PCFC, Fire Testing Technology Limited Co., East Grinstead, UK) under the ASTM 7309-2007a standard and a heating rate of 1 °C/s was selected. The MCC was tested under an atmosphere with a mixture of nitrogen and oxygen in a ratio of 8:2.

### 3.3. Synthesis of (3aR,4S,7R,7aS)-2-(3-(Furan-2-ylmethyl)-3,4-dihydro-2H-benzo[e][1,3]oxazin-8-yl)-3a,4,7,7a-tetrahydro-1H-4,7-methanoisoindole-1,3(2H)-dione (Abbreviated as **o*HPNI-fa***)

**Scheme 3:**
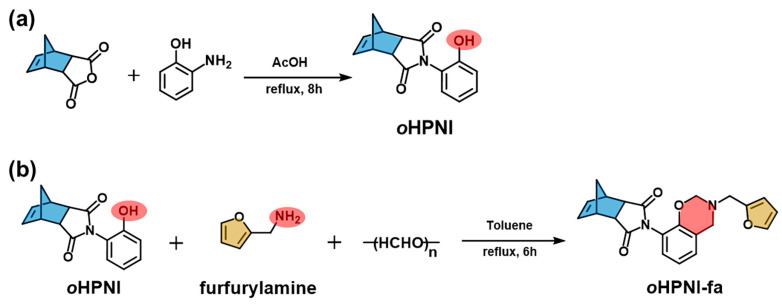
Synthesis of ***o*HPNI** (**a**) and ***o*HPNI-fa** (**b**).

Firstly, into a 250 mL single-neck flask, ***o*HPNI** (2.552 g, 0.010 mol), furfurylamine (0.972 g, 0.010 mol), paraformaldehyde (0.66 g, 0.022 mol), and 120 mL of toluene were added with a magnetic stirrer. The solid was dissolved at 60 °C with the help of magnetic stirring. The entire system was then gradually heated to 110 °C and reacted for 6 h. The reaction solution was rinsed twice with distilled water and once with an aqueous solution of 1 N NaOH after the system had cooled down to room temperature, and then the recrystallization was performed (toluene:ethanol = 1:1). Eventually, 48 h was allocated for drying in a vacuum oven at 50 °C to obtain the pale-yellow crystal product. (yield ca. 76%). The chemical reaction is shown in Scheme 3.

^1^H NMR (400 MHz, Chloroform-*d*) δ 7.43 (dd, J = 4.5, 1.8 Hz, 1H), 7.04–6.87 (m, 2H), 6.82 (dd, J = 7.8, 1.7 Hz, 1H), 6.34 (ddd, J = 5.3, 3.2, 1.9 Hz, 1H), 6.27 (dt, J = 13.9, 2.2 Hz, 2H), 6.18 (d, J = 1.9 Hz, 1H), 4.87 (d, J = 15.8 Hz, 2H), 4.03 (d, J = 5.4 Hz, 2H), 3.90 (d, J = 3.6 Hz, 2H), 3.53–3.39 (m, 4H), 1.84–1.73 (m, 1H), 1.66–1.55 (m, 1H).

^13^C NMR (101 MHz, Chloroform-*d*) δ 176.52, 176.40, 151.26, 149.52, 149.38, 142.70, 134.63, 134.53, 128.57, 127.58, 127.16, 120.83, 120.69, 120.22, 119.43, 110.18, 109.32, 82.34, 81.68, 52.32, 51.93, 49.36, 49.06, 48.12, 48.01, 46.56, 46.07, 45.30.

HR-MS (ESI) (*m*/*z*), [M + H]^+^ calcd for C_22_H_21_N_2_O_4_^+^, 377.1496; found, 377.1494.

Anal. calcd for C_17_H_14_N_2_O_4_: C, 70.20; H, 5.36; N, 7.44. Found: C, 70.17; H, 5.38%; N, 7.42.

## 4. Conclusions

In the current work, a terpolymerizable benzoxazine resin containing both functionalities of norbornene as well as furan group was designed and successfully synthesized. The newly obtained benzoxazine can be regarded as partial bio-based thermosetting resin since one of the raw materials, furfurylamine, is obtained from the natural renewable resource. Even though there are no additional polymerization catalysts or anti-flammable chemicals present, the obtained thermosetting resin still possessed both low temperature polymerization and non-flammable properties. With a *T*_g_ of 287 °C and a T_d5_ temperature of 395 °C, the resultant thermoset also demonstrated excellent thermal stability. As a result, the multifunctional halogen-free thermosetting resin obtained in this study has an infinite potential to replace halogenated polymers in many high-performance industries.

## Figures and Tables

**Table 1:** DSC data and activation energy of benzoxazine monomers.

Monomer	Ea (KJ/mol)	
	Kissinger	Ozawa
**PH-fa**	77.13	81.41
***o*HPNI-a**	117.92	120.51
***o*HPNI-fa**	134.07	135.36

**Table 2:** Thermal and anti-flame characteristics of **poly(*o*HPNI-fa)**.

Sample	*T*_g_ (DMA) (°C)	T_d5_ (°C)	T_d10_ (°C)	Yc (wt.%)	HRC (Jg^−1^K^−1^)	THR (kJg^−1^)
**poly(** ***o*** **HPNI-fa)**	287	395	425	56	56.8	4.9

## Data Availability

Data will be made available on request.

## Supplementary Materials

The following supporting information can be downloaded at: https://www.mdpi.com/article/10.3390/molecules28093944/s1, Figure S1: HR-MS spectrum of ***o*HPNI-fa**; Figure S2: (a) DSC thermograms of **PH-fa** at different heating rates. (b) Representations of the calculation of activation energy for **PH-fa**.; Figure S3: (a) DSC thermograms of ***o*HPNI-a** at different heating rates. (b) Representations of the calculation of activation energy for ***o*HPNI-a**. ^1^H NMR spectrum of **PH-fa** and ***o*HPNI-a** are also included.

## Sample Availability

Samples of the benzoxazine monomer are available from the authors.
