# Supplementary material for: Low-Temperature Terpolymerizable Benzoxazine Monomer Bearing Norbornene and Furan Groups: Synthesis, Characterization, Polymerization, and Properties of Its Polymer
Source: Molecules. 2023 May 7;28(9):3944. doi: 10.3390/molecules28093944 (PMC10179839; doi:10.3390/molecules28093944)

Supporting Information

Low-temperature Terpolymerizable Benzoxazine Monomer Bearing Norbornene and Furan Groups: Synthesis, Characterization, Polymerization and Properties of Its Polymer

Yin Lu [1], Yaliang Peng [1], Yi Yang [1], Jiahao Liu[2] and Kan Zhang [1,*]

[1]Research School of Polymeric Materials, School of Materials Science and Engineering, Jiangsu University, Zhenjiang 212013, China

[2]School of Mechanical Engineering, Jiangsu University, Zhenjiang 212013, China

*: To whom correspondence should be addressed:

Kan Zhang: zhangkan@ujs.edu.cn

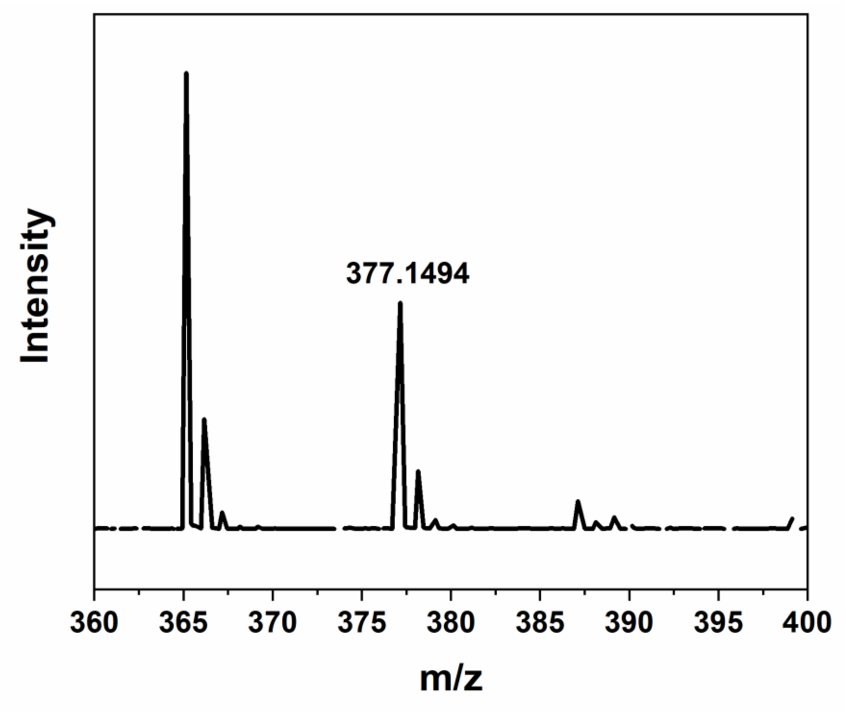

Figure S1. HR-MS spectrum of ***o*HPNI-fa**.

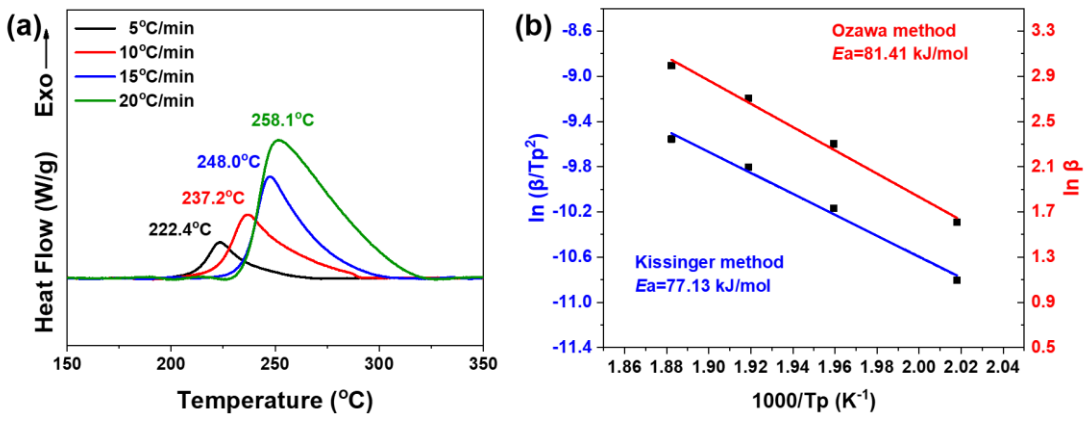

Figure S2. (a) DSC thermograms of **PH-fa** at different heating rates. (b)Representations of the calculation of activation energy for **PH-fa**.

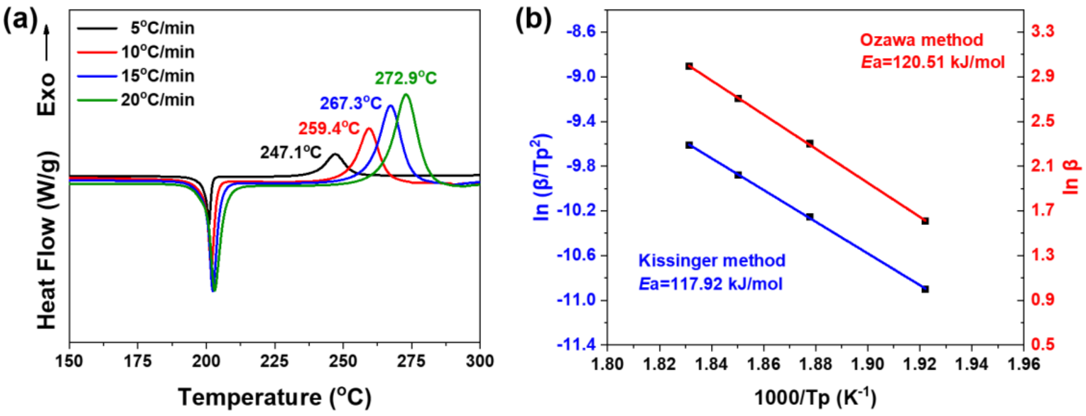

Figure S3. (a) DSC thermograms of ***o*****HPNI-a** at different heating rates. (b)Representations of the calculation of actvation energy for ***o*****HPNI-a**.

^{1}H NMR spectrum of **PH-fa**

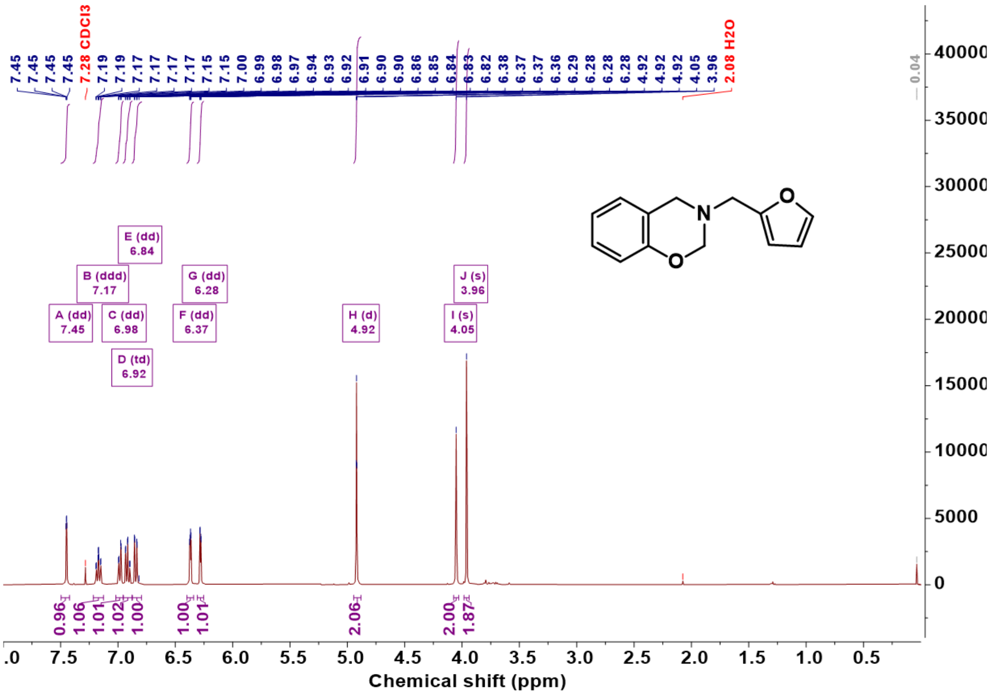

^{1}H NMR spectrum of ***o*HPNI-a**

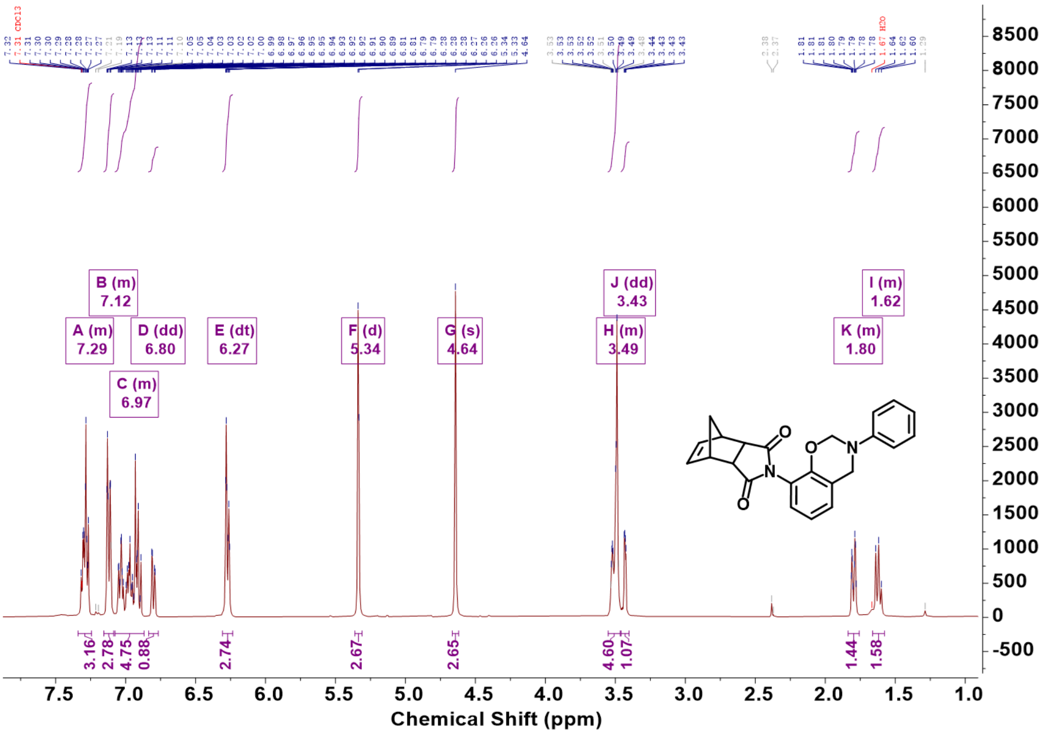

Supplement: Supplementary file 1 [file molecules-28-03944-s001.zip › molecules-2326997-supplementary.pdf]
